# A Strategy for Synthesis of Pathogenic Human Immunoglobulin Free Light Chains in *E. coli*

**DOI:** 10.1371/journal.pone.0076022

**Published:** 2013-09-27

**Authors:** Paola Rognoni, Francesca Lavatelli, Simona Casarini, Giovanni Palladini, Laura Verga, Paolo Pedrazzoli, Giovanna Valentini, Giampaolo Merlini, Vittorio Perfetti

**Affiliations:** 1 Amyloidosis Research and Treatment Center and Biotechnology Research Laboratories, Fondazione IRCCS Policlinico S. Matteo and Department of Molecular Medicine, University of Pavia, Pavia, Italy; 2 Pathologic Unit, Fondazione IRCCS Policlinico S. Matteo, Pavia, Italy; 3 Department of Hematology and Oncology, Section of Oncology, Fondazione IRCCS Policlinico S. Matteo, Pavia, Italy; 4 Department of Biology and Biotechnology “Lazzaro Spallanzani”, University of Pavia, Pavia, Italy; Naval Research Laboratory, United States of America

## Abstract

Monoclonal immunoglobulin light chains are normally synthesized in excess compared to the heavy chain partners and can be detected in serum and urine (“free” LC). Occasionally free LC are *per se* cause of organ toxicity, as in free LC-related disorders. In AL amyloidosis, the most common of these conditions, free LC with peculiar biophysical properties related to their primary structure damage target organs and organize in amyloid fibrils. Unlimited availability of well-characterized free LC is instrumental to investigate the toxic effect of these proteins and to study their interactions with targets. We present a straightforward strategy to obtain recombinant monoclonal free LC by using a bacterial system. These proteins, expressed as inclusion bodies, were subjected to solubilization and refolding procedures to recover them in native form. To minimize differences from the circulating natural LC, full-length recombinant LC were expressed, i.e. complete of variable and constant regions, with the original amino acid sequence along the entire protein, and with no purification tags. The strategy was exploited to generate free LC from three AL amyloidosis patients. After purification, recombinant proteins were biochemically characterized and compared to the natural Bence Jones protein isolated from one of the patients. Results showed that the recombinant free LC were properly folded and formed homodimers in solution, similar to the natural Bence Jones protein used for comparison. Furthermore, as proof of pathogenicity, recombinant proteins formed amyloid fibrils *in vitro*. We believe that the present strategy represents a valuable tool to speed research in free LC-related disorders.

## Introduction

Human immunoglobulin light chains (LC) consist of two domains, each of 100-110 amino acids (12 kDa, approximately), that fold independently of each other: the variable region domain (V_L_), generated via rearrangement of a V to a J gene segment, and the constant region domain (C_L_), of minimal variation. LC can occur in two isotypes, namely κ and λ [1]. The two LC domains have similar compact tertiary structure with a hydrophobic core composed of two twisted β-sheets, which are stabilized by a single intrachain disulfide bond. On the other hand, a cysteine (for a total of 5 cysteines in a single LC molecule) located at the carboxyterminal end, forms an interchain disulfide bond with the immunoglobulin heavy chain. LC are usually synthesized in large excess over heavy chains, and this results in production of “free” LC that can be detected in serum and urine [2]. Kappa free LC are normally monomeric and can be found with various degrees of glycosylation, while lambda free-light chains tend to be dimeric and unglycosylated [3]–[5].

Monoclonal free LC are not only a hallmark of clonal B-cell expansions; these molecules can be responsible of a variety of pathological conditions, including LC deposition disease, acquired Fanconi syndrome, LC cast nephropathy and AL amyloidosis, the most common of systemic amyloidoses and of the free LC-related disorders [6].

In AL amyloidosis, bone marrow plasma cells secrete monoclonal free LC, typically of the λ isotype, that undergo extracellular deposition as fibrils (amyloid) at distant sites [7]. Since amyloid deposition occurs in just a fraction of subjects with monoclonal gammapathy and free LC production, and it recurs in transplanted organs if LC synthesis is not halted by chemotherapy, peculiar molecular features of free LC must be involved in determining protein “amyloidogenicity”. Indeed, the V_L_ gene repertoire of amyloid LC is substantially skewed [8]–[10] and different from the normal one [8], with significant preferential expression of two V_L_ segments, namely *IGLV6-57* [8]–[10] and *IGLV3-1* [8], [10], indicating the existence of amyloid-associated germline gene segments. Furthermore, by the analysis of the relationship between LC gene use and the two most frequent organ involved at diagnosis, kidney and heart, a close association was established between expression of *IGLV6-57* and renal involvement [8]–[10], and between *IGLV1-44* and dominant heart involvement [11]. This evidence provided important insights to understand the bases of amyloid organ tropism, a phenomenon that influences clinical presentation, prognosis and therapeutic options.

It is now widely recognized that the presence of amyloid fibrils alone is not sufficient to explain the cellular and organ damage observed in patients. This aspect is thought to be a common element in all amyloid diseases [12], [13]. In AL amyloidosis, a major clue towards this conclusion came from clinical evidence; variations in the concentration of circulating amyloidogenic free LC are immediately paralleled by concordant and proportional variations of NT-proBNP concentration, a marker of cardiac damage; changes in NT-proBNP concentrations, in turn, reflect rapid improvement or worsening of heart dysfunction, even in the absence of a reduction of cardiac amyloid load, as estimated by echocardiography [14]. These data indicated that cardiac dysfunction is primarily affected by circulating free LC, exerting direct cardiac toxicity. Indeed, *in vitro* studies on animal cultured cells showed alterations upon exposure to LC purified from urine [Bence Jones (BJ) proteins] of AL patients with heart involvement. In particular, a rapid induction of cell toxicity was reported in cultured rat cardiomyocytes and in isolated mouse hearts [15]–[17].

For the above reasons, it is predicted that further insights into the pathogenesis of these disorders may come from studies dealing with the soluble pathogenic molecules; however, discrete amounts of free LC are needed for these investigations, whilst these proteins are frequently found in low concentrations in human fluids [18] and are not always available. Recombinant (r) free LC production in *E. coli* may be a way to provide well-characterized proteins from archive cDNA. However, proteins containing multiple disulfide bonds, such as complete free LC (V_L_+C_L_), are difficult to produce in bacteria, and mainly accumulate in the cytoplasm as inclusion bodies, particles formed by densely packed denatured protein molecules [19]. For this reason, engineering the relevant cDNA with bacteria-derived peptide leader signals that allow for protein translocation into the more favorable, oxidizing environment of the bacteria periplasm is considered the method of choice for LC production [19]. However, potential drawbacks are suboptimal yield, complex isolation and incomplete removal of the peptide leader.

In the present paper we devised a novel, straightforward strategy comprising inclusion body formation as a natural way to obtain consistent amounts of recombinant proteins virtually free of contaminants, with carefully optimized conditions for proper refolding and disulfide bond formation *in vitro*. Given the paramount relevance of the LC structure in the pathogenic process, the strategy was designed to obtain proteins as similar as possible to natural circulating LC: original primary sequence, full-length protein (V_L_ + C_L_), no purification tags (usually consisting of a polyhistidine stretch added to one of the two amino acid terminals of the protein). We here describe free rLC production from two patients with predominant heart amyloidosis and one with exclusive kidney involvement.

## Materials and Methods

### Cloning of complete monoclonal free LC cDNA

Total RNA was extracted from 10^7^ bone marrow mononuclear cells from three AL amyloidosis patients using TRIzol reagent (Life Technologies, Paisley, United Kingdom). Samples were obtained during clinical evaluation, after acquisition of the written informed consent for genetic investigations on patient's material. The study was approved by the IRCCS Policlinico San Matteo Ethics Committee. Monoclonal V_L_ region nucleotide sequences were cloned by an inverse PCR strategy that preserves the original sequence at 5′ and 3′ ends [20]. After sequencing, to obtain the original full-length monoclonal LC (V_L_ + C_L_, approximately 650 bp, from codons +1 to +216), standard RT-PCR was employed using the same marrow RNA, a forward patient-specific primer (dictated by codons +1 to +7 of the monoclonal V_L_ sequence) and a universal reverse C_λ_ carboxyterminal cloning primer, corresponding to the last amino acids of C_L_ (codons +208 to +215, 5′-TGAACATTCTGTAGGGGCCACTGT-3′). In case of κLC expression, the universal 3′ C_κ_ primer must be used: 5'-ACACTCTCCCCTGTTGAAGCT-3'. The PCR fragment was sequenced from both sides, and sequences were submitted to GenBank (*GenBank accession nos.: KC433670, KC433671, KC433672*).

### Construction of the expression vector encoding the recombinant free LC cDNA

**Figure 1:**
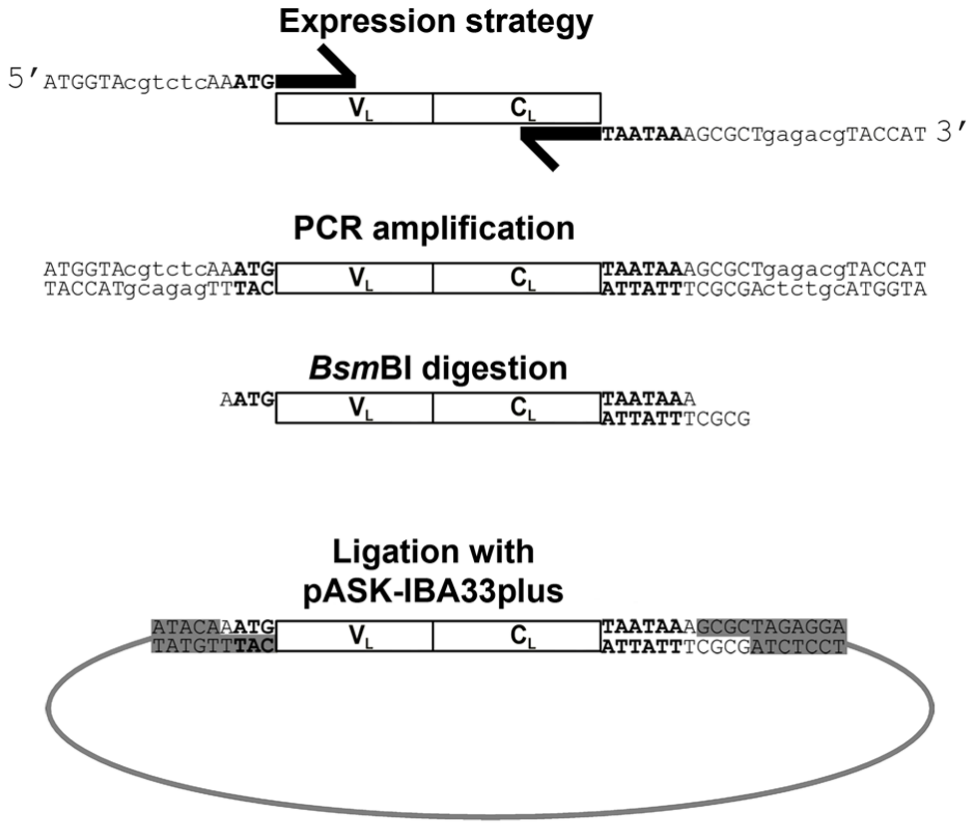
Schematic representation of the expression strategy. Each patient’s full-length monoclonal LC (V_L_ +C_L_) obtained by RT-PCR was re-amplified using primers designed according to the pASK-IBA cloning strategy. Start Met codon in 5′ primer and the two stop codons in 3′ primer were introduced and shown in bold, *Bsm*BI recognition sites are indicated in lower case in both primers. After *Bsm*BI digestion, PCR product was ligated into *Bsa*I/*Bsa*I sites of pASK-IBA33plus vector.

The vector used to express monoclonal complete LC was the pASK-IBA33plus (IBA, Göttingen, Germany), carrying the inducible tetracycline promoter/operator and the ampicillin resistance cassette, originally designed for cytoplasmic protein expression with C-terminal His-tag. The recombinant monoclonal LC (Figure 1) was obtained via PCR reamplification (94°C, 5 sec; 65°C, 45 sec; 72°C, 45 sec; 30 cycles) of the V_L_+C_L_ cDNA, using primers designed according to the pASK-IBA protein cloning strategy, which requires internal *Bsm*BI recognition sites both at the 5′ and 3′ primers (Figure 1). The patient-specific forward primer was: 5′-ATGGTAcgtctcAA**ATG-**patient-specific V_L_ nucleotides-3′, which included *Bsm*BI recognition site (lower case), initiation codon (bold) and patient-specific V_L_ +1 to +22 nucleotides (Figure 1). The universal λ reverse primer was: 5′-ATGGTAcgtctcAGCGCT***TTATTA***TGAACATTCTGTAGGGGCCACTG-3′, which included *Bsm*BI recognition site (lower case), and two stop codons (bold italic) after the last monoclonal LC constant region nucleotide triplet (Figure 1). For κLC expression the following universal reverse primer is adopted: 5′ -ATGGTAcgtctcAGCGCT***TTATTA***ACACTCTCCCCTGTTGAAGCT-3′. After *Bsm*BI digestion, each PCR product was ligated into *Bsa*I/*Bsa*I sites of the expression vector pASK-IBA33plus (Figure 1). Plasmid amplification was carried out into the *E. coli* DH5α cells; insert sequence and reading frame were checked.

### Recombinant free LC expression

*E. coli* BL21(DE3) cells (Merck Chemicals, Nottingham, UK) were transformed with the recombinant LC expression vectors and grown at 37°C in Luria-Bertani medium containing 100 µg/mL ampicillin. When the culture optical density at 600nm reached a value of 0.5–0.6, expression was induced by anhydrotetracycline addition to a final concentration of 0.43 µM. The protein expression was obtained incubating the induced culture at 37°C for 4 h. rLC were found as inclusion body proteins.

### Isolation of inclusion bodies from *E. coli* cells

Typically, *E. coli* cells obtained from 1 L culture were collected by centrifugation (7000 g, 10 min) and resuspended in 70 mL ice-cold buffer (Buffer A: 50 mM TRIS-HCl pH 8.0, 1 mM ethylenediaminetetraacetic acid, 1 mM phenylmethylsulfonylfluoride). The cell suspension was sonicated at 200 W for 6 min with UP200S sonicator (Hielscher, Teltow, Germany), and centrifuged at 30000 g for 15 min at 4 °C. The pellet, containing inclusion bodies, was washed twice with ice-cold Buffer A.

### Solubilization and refolding of monoclonal LC from inclusion bodies

The inclusion bodies were resuspended in 15 mL ice-cold denaturing buffer (Buffer A containing 6 M guanidinium chloride and 5 mM β-mercaptoethanol), incubated under mild agitation at 4 °C for 5 h and centrifuged at 30000 g for 15 min. Refolding of solubilized rLC into their bioactive form was obtained by a dilution method in the presence of reduced and oxidized glutathione [21], [22]. To this end, protein solution containing the solubilized rLC was 20-fold diluted by dripping into an ice-cold refolding-redox shuffle buffer (Buffer A containing 5 mM reduced L-glutathione and 0.5 mM oxidized L-glutathione), under mild stirring. After overnight incubation at 4°C under mild agitation, the protein solution was centrifuged (30000 g, 15 min), and the supernatant was dialyzed overnight against 50 mM TRIS-HCl pH 8.0.

### Recombinant LC purification

The recombinant proteins were purified by a single step procedure, consisting in an anion exchange chromatography on a HiPrep 16/10 Q FF pre-packed column (GE Healthcare, Piscataway, NJ, USA) equilibrated in 50 mM TRIS-HCl pH 8.0. Refolded rLC obtained from inclusion bodies were applied to the column, on an AKTÄPurifier® FPLC system (GE Healthcare), and eluted by the use of a 0 to 1 M NaCl linear gradient. The homogeneity of the protein preparation was assessed by 12% SDS-PAGE and by immunoblotting, following standard procedures. The protein concentration was evaluated using Pierce BCA Protein Assay Kit (Thermo Scientific, Rockford, IL, USA); bovine serum albumin was used as standard.

### Size exclusion chromatography

The purified rLC were subjected to size exclusion chromatography on an HiLoad 16/60 Superdex 75 pre-packed column (GE Healthcare) equilibrated in 50 mM sodium phosphate pH 7.4. Samples (50 µg) were applied to the column on an AKTÄPurifier® FPLC system and eluted with the equilibration buffer at 0.6 mL/min, monitoring at 280 nm. Column was calibrated with the following proteins: conalbumin (75.0 kDa), ovalbumin (44.0 kDa), carbonic anhydrase (29.0 kDa), ribonuclease A (13.5 kDa), aprotinin (6.5 kDa) (GE Healthcare).

### Quantification of thiols in free LC

Free thiol content of purified proteins was quantified by the Measure-iT™ Thiol Assay kit (Invitrogen). The fluorescence of samples (tested from 5 to 30 pmol) was determined at λ_ex_ =  490 nm and λ_em_ =  520 nm in clear 96-well plates on an Infinite F200Pro plate reader (Tecan, Männedorf, Switzerland).

### Circular dichroism spectroscopy

Far-UV circular dichroism (CD) measurements were performed with a Jasco J-700 spectropolarimeter (Jasco Europe, Cremella, Italy) using a quartz cuvette with a path length of 1 mm. Scans were conducted from 250 to 200 nm at a speed of 20 nm/min with a spectral band width of 2 nm, a sensitivity of 20 millidegrees and response time of 1 sec. CD spectra measurements were performed at 25°C in 50 mM sodium phosphate pH 7.4 and pH 5.0, and represent the average of 10 scans. The protein concentration was 0.4 mg/mL. The α-helical and β-sheet content was calculated with K2D, CDSSTR and CONTIN software applications [23]–[25].

### Analysis of rLC by MALDI-TOF mass spectrometry

rLC were dialyzed overnight against 50 mM ammonium bicarbonate and reduced by addition of dithiothreithol (final concentration 10 mM, 5 min at 95°C, 45 min at 25°C). Intact mass determination was performed immediately after reduction by MALDI-TOF mass spectrometry, on a Micromass Micro MX instrument (Waters, Milford, MA, USA), operated in positive-ion, linear mode, over the 7500–30000 *m/z* range. Samples (protein concentration: 0.25–0.5 mg/mL) were co-crystallized onto target plate with the matrix α-cyano-4-hydroxycinnamic acid (CHCA, 1∶1 v/v). External calibration was performed using a mixture of protein standards (cytochrome C, trypsinogen, myoglobin, all from Sigma Aldrich, Steinheim, DE). Spectra were processed and analyzed using MassLynx software (Waters). Theoretical masses of the intact proteins (from bone marrow-deduced LC sequences, and from the same sequences upon addition of a methionine residue at the N-terminus) were calculated with the on-line software tool MS-Digest (Protein Prospector, University of California, San Francisco, CA, USA).

### AL-2 patient’s BJ protein purification

BJ protein from AL-2 patient (AL-2 BJ) was purified from 24 h urine collection. The urine, immediately made 0.1% w/v NaN_3_ to reduce degradation phenomena and bacterial proliferation and centrifuged at 3000 g for 30 min to remove cells and insoluble material, was subjected to ammonium sulfate precipitation (65% of saturation) and incubated overnight at 4 °C. The precipitate was collected by centrifugation (3000 g, 30 min) and dialyzed overnight against 10 mM sodium phosphate, pH 7.0. All steps were performed at 4°C.

Dialyzed proteins were applied to the pre-packed column HiPrep 16/10 Q FF (GE Healthcare), equilibrated in 10 mM sodium phosphate, pH 7.0 and eluted using a 0 to 1 M NaCl linear gradient. The homogeneity of the isolated species was assessed by 12% SDS-PAGE and immunoblotting. The protein concentration was evaluated using Pierce BCA Protein Assay Kit; bovine serum albumin was used as standard.

### In Vitro Fibril Formation of rLC and AL-2 BJ

For fibril formation assays, 1 mg/mL LC samples were prepared in 10 mM TRIS-HCl pH 7.0, filtered (0.22 µm) and incubated at 60°C rocking at 250 rpm on a Innova 43R shaker (New Brunswick Scientific, Edison, NJ) for up to 72 h [26]. Aliquots (10 µL) were removed at various time points, added to a blank solution of 20 µM Congo-red (Fisher Scientific, Loughborough, UK) in 25 mM PBS buffer at pH 7.4 and left incubated for 30 min at room temperature. Fibrils formation was monitored by recording the UV spectrum between 400 and 700 nm on an Infinite M200Pro spectrophotometer (Tecan, Männedorf, Switzerland). Congo-red binding was calculated according to Klunk and coworkers [27].

### Electron microscopy of amyloid fibrils

After 72 h [26], 10 µL samples from *in vitro* fibrillogenesis (see above), were applied to carbon-coated Formvar nickel grid (200 mesh) (Electron Microscopy Sciences, Washington, PA, USA). Samples were allowed to sediment on the carbon film for 15 min and the negative staining was performed with 10 µL of 2% w/v uranyl acetate solution (Electron Microscopy Sciences). After draining off the excess of staining solution by means of a filter paper, the specimens were transferred to the electron microscope for examination, using a Philips CM12 transmission electron microscope, operating at 80 kV [28]. Electron micrographs of negatively stained samples were photographed on Kodak film. Magnification 60000 x.

## Results and Discussion

In this paper we describe a strategy to obtain significant amounts of human immunoglobulin free LC by the use of a bacterial cell factory. By means of this novel procedure, it is possible to obtain recombinant proteins that closely reproduce the natural pathogenic LC, in this case from AL amyloidosis, a disorder characterized by altered, unstable, toxic free LC whose primary structure is recognized to be a determinant in the pathogenic process.

### Monoclonal LC cloning and sequencing

In order to clone the V_L_, we adopted a consolidated inverse PCR procedure to preserve the original nucleotide sequence at the extremities of the V_L_ region [8], [11]. We consider this point of great relevance given the role in protein folding, and consequently in the function of the LC, of the first amino acids of V_L_ [29]. Somatic mutations leading to amino acid changes in these areas of the molecule are also quite common in amyloidosis [8], [11]. Although we consider the use of inverse PCR preferable, alternative V_L_ amplification methods could be used without introducing modifications to the expression strategy.

Based on amino terminal sequence information, clone-specific primers were designed for standard RT-PCR to obtain the complete full-length LC sequence (V_L_+C_L_). Sequencing of the cloned cDNA fragments showed that LC were potentially functional, being devoid of stop codons or frameshift mutations. LC derived from somatic mutation of different germline gene segments, *IGLV1-44*

(AL-1 and AL-3) and *IGLV1-51* (AL-2). No changes from the reported λ C_L_ sequences were observed. In order to recreate proteins that were most similar to the natural circulating LC, and at variance from previous studies focused on truncated proteins [30]–[32], we synthesized the complete LC. Indeed, the relevance of working with the whole LC is becoming to be appreciated [33], [34], given the important influence of the constant region in the thermodynamic properties of the LC and in the process of amyloid formation [33]. Indeed, it has been shown that full-length LC are more likely to initiate aggregation during unfolding and provide a template for the V_L_ deposition [33].

### Expression vector construction and rLC production

The PCR fragment encoding the full-length cDNA of the amyloid LC was cloned into the expression vector pASK-IBA33plus (Figure 1), stop codons were introduced at 3′ via PCR to express recombinant proteins free of the C-terminal His-tag. By means of this strategy (Figure 1), the rLC were predicted to be identical to the original one, with the last amino acid consisting of the invariant Ser216, which follows the Cys215 involved in the interchain disulfide bond, with just a single amino acid addition consisting of the 5′ Met encoded by the initiating codon ATG. This extra codon was included into the expression primer to allow transcription (Figure 1). However, Met is expected not to be detrimental for the biochemical and biophysical properties of the protein, since methionine is a small and uncharged amino acid residue, in a position that in LC is typically exposed to solvent and not involved in interactions with other amino acids [1]. The preservation of Cys215, at the end of C_L_, is relevant for the process of dimerization [1], a natural tendency of the free LC (see below).

**Figure 2:**
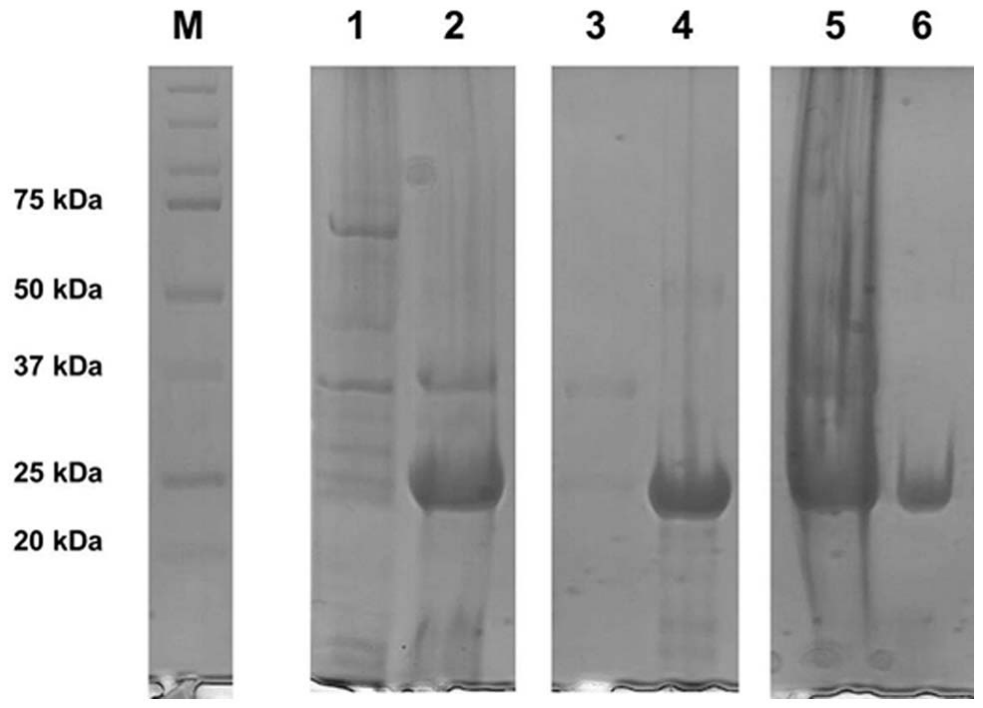
Different steps of rAL-1 renaturation from inclusion bodies . Protein samples were subjected to reducing 12% SDS-PAGE and stained with Coomassie Brilliant Blue. M: molecular mass markers; lane 1, supernatant of bacteria sonication; lane 2, pellet of cell sonication, containing inclusion bodies; lane 3, pellet of inclusion bodies solubilization; lane 4, supernatant of inclusion bodies solubilization; lane 5, pellet of recombinant protein refolding; lane 6, supernatant of recombinant protein refolding. A significant proportion of the expressed protein was soluble after refolding from inclusion bodies.

The rLC were expressed in BL21(DE3) cells. Protein production was monitored by SDS-PAGE analysis after induction. At the best time point (4 h of induction at 37 °C), after bacteria collection and sonication, the homogenate was centrifuged, supernatant (containing soluble proteins) and pellet were analyzed for recombinant protein content. As expected, recombinant products were recovered in the pellet fraction as inclusion bodies (Figure 2, lane 2). This feature was advantageous for purification (see below).

### Recombinant LC solubilization and refolding

rLC were solubilized from inclusion bodies using reducing and denaturing conditions, working at low temperature, to prevent bacterial proteolysis. As shown in Figure 2, lane 4, the recombinant protein was found in the supernatant, indicating that the denaturing conditions allowed the total solubilization of the rLC.

To obtain rLC refolding with concomitant disulfide bond formation, a key step in the recovery of bioactive proteins from inclusion bodies, we set up a glutathione redox-shuffle [21], [22].

condition, which involved an appropriate mixture of oxidized and reduced thiol reagents (reduced/oxidized L-glutathione, ratio 10/1 mM). A similar approach was successfully used for immunoglobulin Fab [35], [36]. After refolding and centrifugation, supernatant was subjected to 12% SDS-PAGE (Figure 2, lane 6). Nearly 40% of total rLC refolded, as estimated by visual inspection (Figure 2, lanes 5 and 6), a typical result for the glutathione redox-shuffle [21], [22], [35]–[37]. Data obtained from 3 different preparations indicated that the final yield of refolded rLC was approximately 35 mg of protein per liter of bacterial culture for rAL-2, whereas approximately 24 mg/liter and 20 mg/liter were the values for rAL-1 and rAL-3, respectively.

### Recombinant LC purification

**Figure 3:**
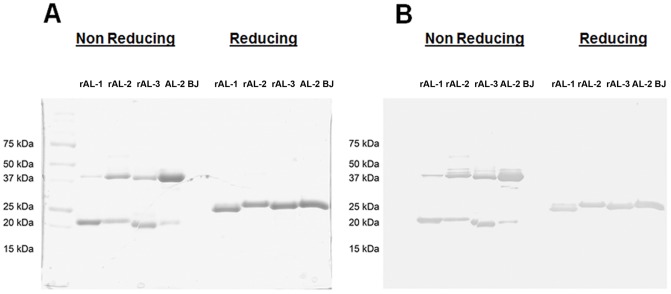
SDS–PAGE analysis of the purified rLC. Coomassie Brilliant Blue staining (A) and western blotting (B) analysis of purified rLC compared to AL-2 Bence Jones protein (AL-2 BJ), purified from urine. Protein samples (5 µg loaded) were resolved on 12% SDS-PAGE run under non reducing and reducing conditions. Western blot analysis was performed using a rabbit anti-human λ LC antiserum as primary antibody (Dako Cytomation, Glostrup, Denmark), revealed by a goat anti-rabbit conjugated with horseradish peroxidase (Dako Cytomation), probed with diaminobenzidine (DAB) substrate. Molecular mass markers are shown in the left lane. The proteins presented the typical dimeric-monomeric species of free LC. Different degrees of protein denaturation are responsible of the minor differences in electrophoretic migration of monoclonal free LC (A), a common finding in SDS PAGE running under denaturing conditions of these proteins.

Refolded rLC were purified to homogeneity by a single step anion-exchange chromatography. Figure 3 shows 12% SDS-PAGE of the 3 purified rLC, with the AL-2 BJ for comparison, run under non-reducing and reducing conditions and stained with Coomassie Brilliant Blue (Figure 3A) or analyzed by western blotting with anti-human λ LC antiserum (Figure 3B). LC migrated as single bands of apparent molecular mass of approximately 25 kDa in the presence of reducing agents, whereas they showed two major bands of apparent molecular mass of approximately 40 and 20 kDa under non-reducing conditions. These data were in agreement with the dimeric and monomeric species of rLC, and were supported by the similar electrophoretic profile observed for AL*-*2 BJ purified protein run in parallel (Figure 3).

### Recombinant LC biochemical and functional characterization

**Figure 4:**
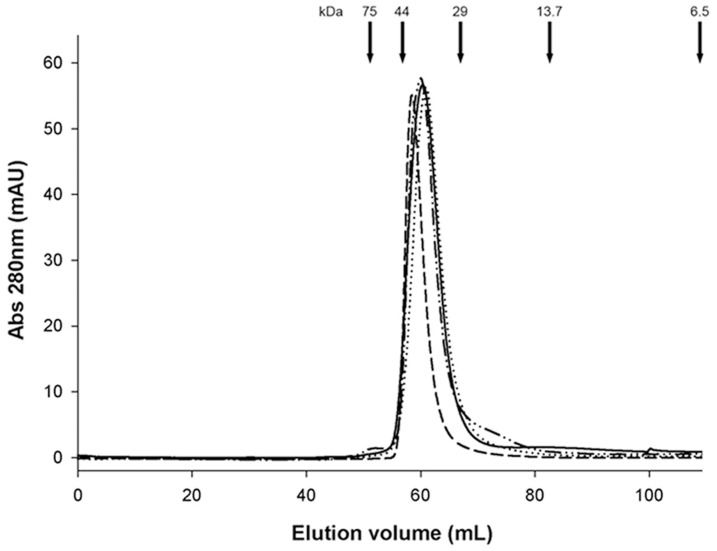
Size exclusion chromatography of rLC. rAL-1 (dotted), rAL-2 (short dashes), rAL-3 (dash-dot-dot) compared with AL-2 BJ protein (solid). Conalbumin (75.0 kDa), ovalbumin (44.0 kDa), carbonic anhydrase (29.0 kDa), ribonuclease A (13.5 kDa), aprotinin (6.5 kDa) were used as molecular mass standards. Virtually identical elution profiles were observed.

The assessment of the oligomeric state of the rLC was performed by size exclusion chromatography. The natural AL-2 BJ protein was also run as control. Each protein eluted from the column as a single main peak, symmetric in shape (Figure 4). Peaks were at a position of approximately 40–44 kDa, in agreement with the expected value for the dimeric form of LC.

In order to test the redox state of cysteines in refolded rLC and isolated AL-2 BJ, free thiol content was determined by means of a highly sensitive fluorimetric assay. Results showed no detectable free thiols in the three rLC and in the natural BJ protein (<0.004 pmol per pmol LC), as expected for LC found in a dimeric state, and in agreement with the size exclusion chromatographic analyses (Figure 4).

**Figure 5:**
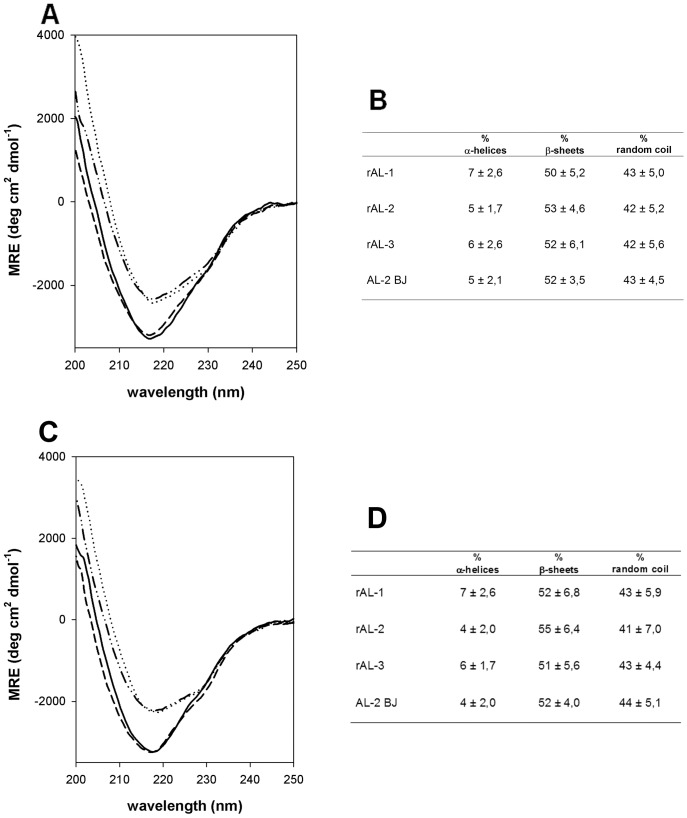
Circular dichroism analyses. Far-ultraviolet CD spectra (panel A, pH 7.4; panel C, pH 5.0) of purified rAL-1 (short dashes), rAL-2 (dotted), rAL-3 (solid) compared with the spectrum of patient AL-2 BJ protein (dash-dot-dot). Each CD spectrum represents the average of ten scans. Data are shown as mean residue ellipticity (MRE) as a function of wavelength. Panels B and D report the secondary structure content of the analyzed proteins (at pH 7.4 and 5.0, respectively), calculated with K2D, CDSSTR and CONTIN software applications. Data are expressed as mean ± sd. Spectra were virtually identical for rAL-2 and the corresponding natural BJ (panels A and C), overlapping for the other two rLC (94% amino acid identity), and display the minimum at around 218 nm, characteristic of β-sheets. Proteins showed very similar percentage of secondary structures.

Figure 5 reports the free LC secondary structure patterns investigated by CD spectroscopy at the pH of serum (pH 7.4, panels A and B) and urine (pH 5.0, panels C and D). Spectra profiles typical of the immunoglobulin LC fold were observed (panels A and C), characterized by the high content of β-sheets of the two structural domains (panels B and D). In analogy to natural free LC, that are stable in serum and urine, the synthetic free LC were capable to preserve their secondary structures at different pH (panels A and C, respectively). In the whole, these data documented comparable and proper folding, and indicated that the expression strategy led to the bioactive native conformation of the rLC.

**Figure 6:**
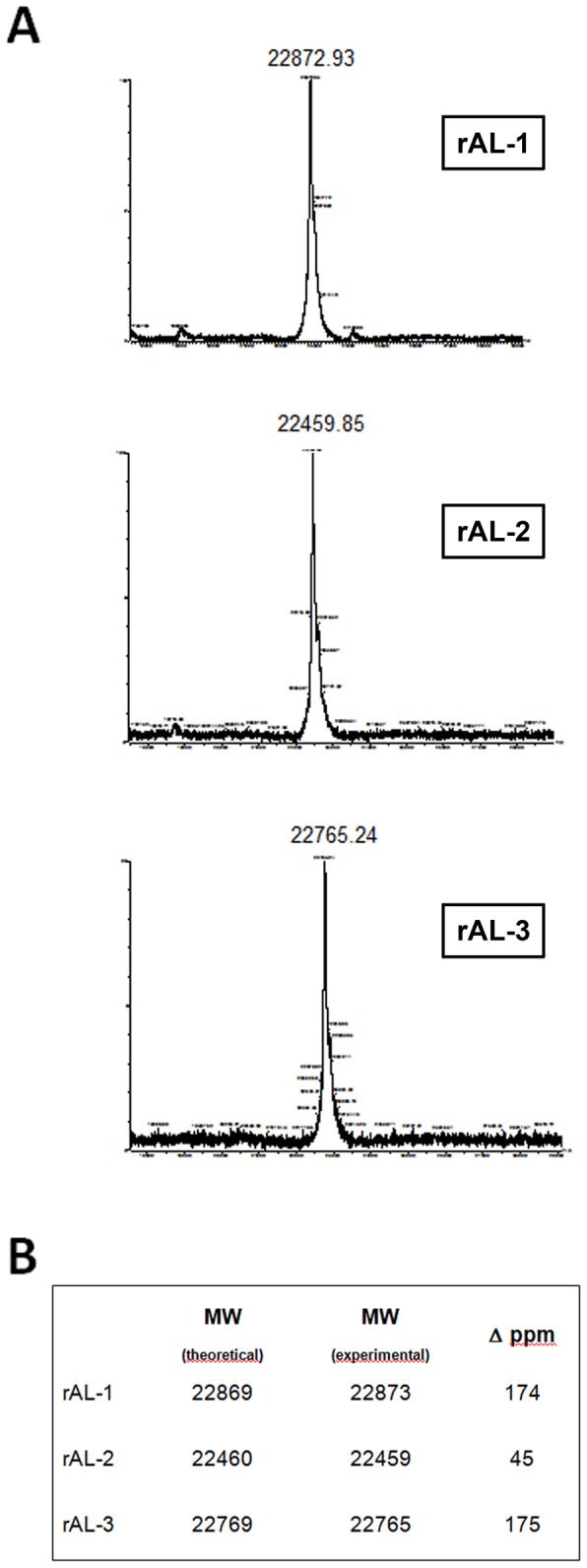
Intact mass determination of purified rLC by MALDI-TOF mass spectrometry. Panel A shows the peaks corresponding to the single charged ions. Panel B shows the calculated (including the presence of an N-terminal methionine) and the observed intact masses of each LC. The error (ppm) from the calculated mass is reported. The results are consistent with the predicted molecular masses.

The molecular masses of purified rLC were assessed by linear MALDI-TOF mass spectrometry. Figure 6A shows the singly charged forms of the full-length rLC; the observed masses were consistent with complete reduction of the disulfide bonds. A mass shift of approximately 131 Da from the theoretical mass was observed in all rLC (Figure 6B), compatible with the presence of the additional methionine residue derived from the transcription codon (see above).

Then, biochemical characterization indicated that the recombinant proteins exhibited the typical properties of immunoglobulin LC, and that the rAL-2 was virtually indistinguishable from the relative BJ protein (Figure 4 and 5). Moreover, rLC proteins were arranged as dimers in solution (Figure 4). This latter feature added further evidence of proper folding, since it is known that the capability of immunoglobulin LC of forming dimers depends essentially on the correct formation of protein’s secondary structure [1].

**Figure 7:**
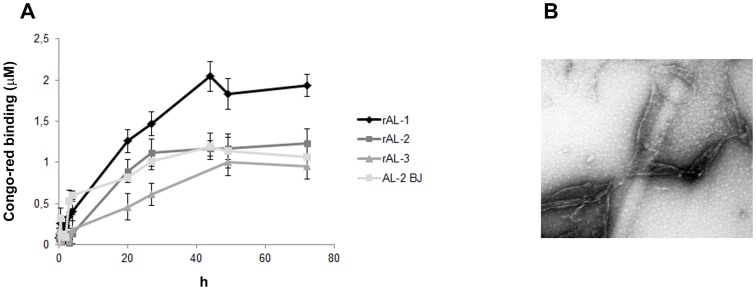
rLC *in vitro* fibrillogenesis. Congo-red binding profiles and electron microscopy. Panel A reports Congo-red binding patterns during fibrillogenesis. rAL-2 and AL-2 BJ revealed similar binding profiles. Panel B shows electron microscopy of typical amyloid fibrils generated *in vitro* by the rLC (fibrils from rAL-2 are shown). Magnification is 60000 x.

As proof of functionality and conserved pathogenic property, we therefore tested the ability of rLC to generate amyloid fibrils *in vitro*. Again, the natural AL-2 BJ was used as control. Congo-red binding assays (Figure 7A) showed typical patterns consistent with fibril formation. rAL-2 and AL-2 BJ showed very similar Congo-red binding profiles (Figure 7A). Electron microscopy of materials from the 72 h end point [26] demonstrated the presence of amyloid fibrils in all cases (Figure 7B, rAL-2).

In conclusion, we developed a procedure that allowed to obtain, through a bacterial system and in a short period of time (about 3 weeks from RNA extraction), significant quantities of free LC (20-35 mg/liter of culture, depending on the protein) from three patients with AL amyloidosis, a disease typically characterized by LC instability and toxicity. The protein expression in the form of inclusion bodies made purification easy, reducing the number of chromatographic steps generally required to obtain homogeneous protein preparations. Refolding yield from inclusion bodies was noteworthy, providing pathogenic free-LC virtually identical to the natural BJ proteins, capable of amyloid fibril formation *in vitro*, and in quantities suitable for most *in vitro* applications.
